# Recent advances in understanding the biomechanics of patellofemoral pain

**DOI:** 10.1186/1757-1146-4-S1-I3

**Published:** 2011-05-20

**Authors:** Kay M Crossley

**Affiliations:** 1 University of Melbourne, Victoria, Australia

## 

While much of the major clinical and research interest in patellofemoral pain (PFP) has been targeted to the local knee muscles, it has long been recognized that proximal (hip and pelvis) biomechanics may be impaired in people with PFP. This clinical observation has led to recent research in pelvis and hip muscle contributions to PFP. Hip muscles (particularly the abductors and external rotators) are important in maintaining an optimal lower limb alignment during weight bearing activities. A growing body of contemporary evidence indicates that hip muscle function is compromised in PFP. This is highlighted by a recent systematic review, which found strong evidence for deficits in hip muscle strength (abduction, external rotation, extension) in women with PFP compared to uninjured controls. We have observed a delayed onset of gluteus medius electromyographic (EMG) activity in people with PFP compared to healthy controls, confirming earlier findings. Reduced strength or neuromotor control of these hip muscles may be associated with an increase in hip internal rotation and adduction, with deleterious consequences at the knee. Notably, altered proximal biomechanics (hip and pelvis) can influence local patellar alignments and joint stress. Thus, the available evidence supports altered proximal biomechanics as an important feature of PFP, and has provided impetus for contemporary clinical management, favouring hip muscle retraining. However, there is a dearth of clinical trials investigating the clinical efficacy of hip muscle retraining in PFP. Similar to vasti muscle dysfunction, individuals with hip muscle dysfunction form a subgroup of people with PFP. Interventions designed to enhance hip muscle function are likely to benefit patients with PFP. Further scientific evidence is required to confirm the role of hip muscle dysfunction in the development and management of PFP.

